# Crimean-Congo Hemorrhagic Fever Virus Diversity and Reassortment, Pakistan, 2017–2020

**DOI:** 10.3201/eid3004.231155

**Published:** 2024-04

**Authors:** Massab Umair, Zaira Rehman, Shannon Whitmer, Melissa Mobley, Ammad Fahim, Aamer Ikram, Muhammad Salman, Joel M. Montgomery, John D. Klena

**Affiliations:** National Institutes of Health Pakistan, Islamabad, Pakistan (M. Umair, Z. Rehman, A. Ikram, M. Salman);; Centers for Disease Control and Prevention, Atlanta, Georgia, USA (S. Whitmer, M. Mobley, J.M. Montgomery, J.D. Klena);; The Indus Hospital and Health Networks, Karachi, Pakistan (A. Fahim)

**Keywords:** Crimean-Congo hemorrhagic fever virus, reassortment, vector-borne infections, viruses, zoonoses, Pakistan

## Abstract

Sporadic cases and outbreaks of Crimean-Congo hemorrhagic fever (CCHF) have been documented across Pakistan since 1976; however, data regarding the diversity of CCHF virus (CCHFV) in Pakistan is sparse. We whole-genome sequenced 36 CCHFV samples collected from persons infected in Pakistan during 2017–2020. Most CCHF cases were from Rawalpindi (n = 10), followed by Peshawar (n = 7) and Islamabad (n = 4). Phylogenetic analysis revealed the Asia-1 genotype was dominant, but 4 reassorted strains were identified. Strains with reassorted medium gene segments clustered with Asia-2 (n = 2) and Africa-2 (n = 1) genotypes; small segment reassortments clustered with the Asia-2 genotype (n = 2). Reassorted viruses showed close identity with isolates from India, Iran, and Tajikistan, suggesting potential crossborder movement of CCHFV. Improved and continuous human, tick, and animal surveillance is needed to define the diversity of circulating CCHFV strains in Pakistan and prevent transmission.

Crimean-Congo hemorrhagic fever (CCHF) is a tickborne hemorrhagic disease caused by CCHF virus (CCHFV) and has a 30% fatality rate (*1*). CCHF was initially reported in 1940 in Crimea; identical disease manifestations were reported in the Democratic Republic of the Congo during the late 1960s (*2*,*3*). The virus comprises a tripartite RNA genome with large (L), medium (M), and small (S) segments and noncoding regions at the 5′ and 3′ termini. The coding regions are critical for virus polymerase activity, transcription, replication, and packaging. The L segment encodes an RNA-dependent RNA polymerase, the M segment encodes a glycoprotein precursor, and the S segments encode a nucleocapsid protein (*4*). The primary vectors for CCHFV transmission are *Hyalomma* ticks; domestic ruminant livestock and wild animals are amplifying hosts (*5*).

CCHF is considered a serious global public health threat because of its widespread geographic distribution in Asia, Africa, Europe, and the Middle East and because no reliable treatment options or vaccines are available. Increased CCHF incidence has been observed in some CCHF-endemic regions in Asia over the past decade (*6*). In Africa, southern and eastern Europe, the Middle East, India, and Asia, ≈10,000–15,000 CCHFV infections occur each year. Most infections are subclinical or unrecognized sporadic cases or epidemics in CCHF-endemic regions; however, subclinical infections are frequently not reported and are thought to be a source of disease transmission (*7*). Hospital-acquired infections are another transmission route and are typically symptomatic (*8*). Factors resulting in severe CCHFV infections are unknown, but polymorphisms in toll-like receptors 8, 9, and 10 of the innate immune system correlate with disease severity (*9*–*11*).

In Asia, Pakistan reports the fourth highest number of human CCHF cases, preceded by Turkey, Russia, and Iran (*12*). Pakistan witnessed its first CCHF outbreak in 1976; since then, consistent sporadic outbreaks have occurred (*13*). Livestock maintenance practices in rural regions of the country, as well as the nomadic lifestyle on the border of Pakistan and Afghanistan, have likely favored the spread of CCHFV via infected ticks. Cattle herd movement is unrestricted in the border areas of Pakistan, warranting careful surveillance measures. Analysis of virus phylogeny is crucial for identifying novel treatment options and new vaccines and determining the broad extent of CCHFV genetic recombination and reassortment (*14*,*15*). We conducted phylogenetic analyses of whole-genome sequences of CCHFV isolated from patients in Pakistan and evaluated the genomic diversity of circulating CCHFV.

## Material and Methods

The National Institutes of Health Pakistan (NIHP) in Islamabad collected CCHFV samples from suspected CCHF cases across the country that were sent for confirmation. During January 2017–December 2020, a total of 795 samples (289 samples in 2017, 224 in 2018, 280 in 2019, and 2 in 2020) from suspected CCHFV cases were tested at NIHP. Virus RNA was extracted from blood samples by using the QIAamp Viral RNA Mini Kit (QIAGEN, https://www.qiagen.com), and real-time PCR was performed by using a RealStar CCHFV RT-PCR Kit (altona Diagnostics, https://www.altona-diagnostics.com) according to manufacturers’ instructions. We selected a subset of CCHFV-positive samples that had a cycle threshold (Ct) <26 for whole-genome sequencing. The study was approved by the NIHP Institutional Review Board.

### Whole-Genome Sequencing of CCHFV

We treated extracted RNA with RNase-free DNase (Roche, https://www.roche.com) and prepared next-generation sequencing libraries by using a TruSeq RNA Access Library Prep Kit (Illumina, https://www.illumina.com) with CCHFV-specific enrichment oligonucleotides for samples that had Cts >26 or for samples that yielded a partial genome without CCHFV enrichment. For all other samples, we prepared libraries using the NEBNext Ultra II Directional RNA Library Prep Kit (New England Biolabs, https://www.neb.com). We sequenced libraries by using either an Illumina MiSeq or MiniSeq (high output 2 × 150 cycles) instrument. We de novo assembled CCHFV genomes by using SPAdes version 3.14.0 (parameter -k auto; https://github.com/ablab/spades). To improve genome coverage, we analyzed contigs by using BLAST (https://blast.ncbi.nlm.nih.gov) to identify the most closely related reference sequences. We mapped reads 3 times to the most closely related CCHFV genomes by using in-house scripts consisting of quality trimming (parameters: printseq-lite -min_qual_mean 25 -trim_qual_right 20 -min_len 50), read mapping (bwa-mem2 software, https://github.com/bwa-mem2), and PCR deduplication (Picard MarkDuplicates, http://picard.sourceforge.net). We called consensus genomes by using Geneious version 10 (https://www.geneious.com) (threshold = 0%, assign quality = total, minimum coverage >2). We inferred evolutionary history by using all available full-length CCHFV genomes from GenBank and RAxML software (https://github.com/amkozlov/raxml-ng) (parameters for tree generation: -m GTRGAMMA -p $RANDOM -f a -x $RANDOM -N 1000); bootstrap support was provided by 1,000 replicates. We deposited CCHFV genomes from this study into GenBank (accession nos. OM162027–130).

### Phylogenetic Analysis

We downloaded reference genomes of all known CCHFV genotypes from GenBank. We conducted BLAST searches of the complete S, M, and L segment sequences and downloaded closely related sequences from GenBank. We performed multiple sequence alignment by using MAFFT (https://mafft.cbrc.jp) and maximum-likelihood analyses for each segment by using IQ-TREE (http://www.iqtree.org) with 1,000 bootstrap replicates. We visualized and annotated the phylogenetic tree by using FigTree v1.4.4 (https://github.com/rambaut/figtree/releases).

## Results

**Figure 1:**
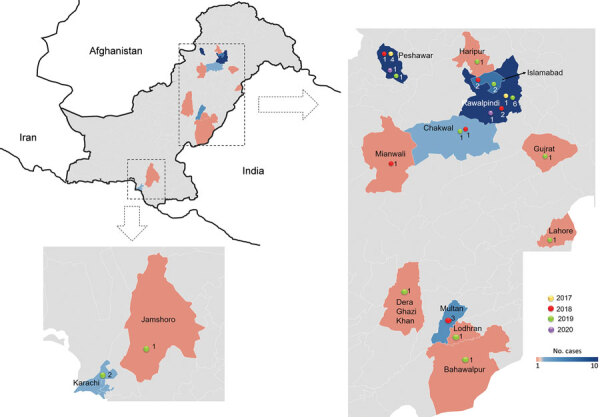
Locations of Crimean-Congo hemorrhagic fever cases in study of virus diversity and reassortment, Pakistan, 2017–2020. Main maps indicate the 2 regions in Pakistan with positive cases. Shading indicates provinces that had 1–10 cases. Inset map shows Pakistan and borders with Afghanistan, India, and Iran.

**Figure 2:**
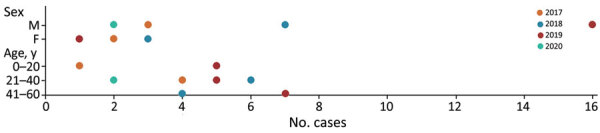
Sex and age distribution of patients with Crimean-Congo hemorrhagic fever in study of virus diversity and reassortment, Pakistan, 2017–2020. Colored dots indicate the number of Crimean-Congo hemorrhagic fever cases each year according to patient sex and age groups.

During 2017–2020, a total of 795 samples were referred to NIHP for CCHFV laboratory diagnosis; 75 samples were CCHFV positive by using quantitative reverse transcription PCR (25 samples collected in 2017, 20 in 2018, 28 in 2019, and 2 in 2020). A Ct <26 was observed in 36 (47%) of those samples, which were then successfully sequenced. The sequenced samples were collected from patients residing in 14 districts; most (n = 21) resided in Punjab Province, followed by Khyber Pakhtunkhwa Province (n = 8), Islamabad (n = 4), and Sindh Province (n = 3) (Figure 1). Demographic and clinical data for 34 (94%) cases were available; 22 (82%) case-patients were male and 6 (18%) female. The mean age of CCHF patients was 35 (SD +15; range 5–60) years (Figure 2). The most common clinical signs and symptoms were fever (34/34 [100%]), hemorrhage (22/34 [65%]) and myalgia (14/34 [41%]). Among hemorrhagic patients, most had hematemesis (9/22 [41%]), followed by gum bleeding (06/22 [27%]), melena (4/22 [18%]), hematuria (2/22 [9%]), and vaginal bleeding (1/22 [5%]). Of the 34 CCHF cases, 12 patients died (35% case-fatality rate) (Table 1).

**Table 1:** Characteristics of confirmed cases of Crimean-Congo hemorrhagic fever in Pakistan, 2017–2020

Characteristics	No. (%) cases			
	2017, n = 5	2018, n = 10	2019, n = 17	2020, n = 2
Clinical signs				
Fever	5 (100)	10 (100)	17 (100)	2 (100)
Hemorrhage	4 (80)	6 (60)	11 (65)	1 (50)
Myalgia	3 (60)	4 (40)	7 (41)	0
Nausea	0	2 (20)	3 (18)	0
Vomiting	0	2 (20)	3 (18)	1 (50)
Headache	0	2 (20)	2 (12)	0
Hemorrhage types				
Melena	1 (20)	1 (10)	1 (6)	1 (50)
Gum bleeding	1 (20)	1 (10)	4 (24)	0
Hematemesis	2 (40)	2 (20)	5 (29)	0
Hematuria	0	1 (10)	1 (6)	0
Vaginal bleeding	0	1 (10)	0	0
Patient outcome				
Survived	4 (80)	8 (80)	9 (53)	1 (50)
Died	1 (20)	2 (20)	8 (47)	1 (50)

**Figure 3:**
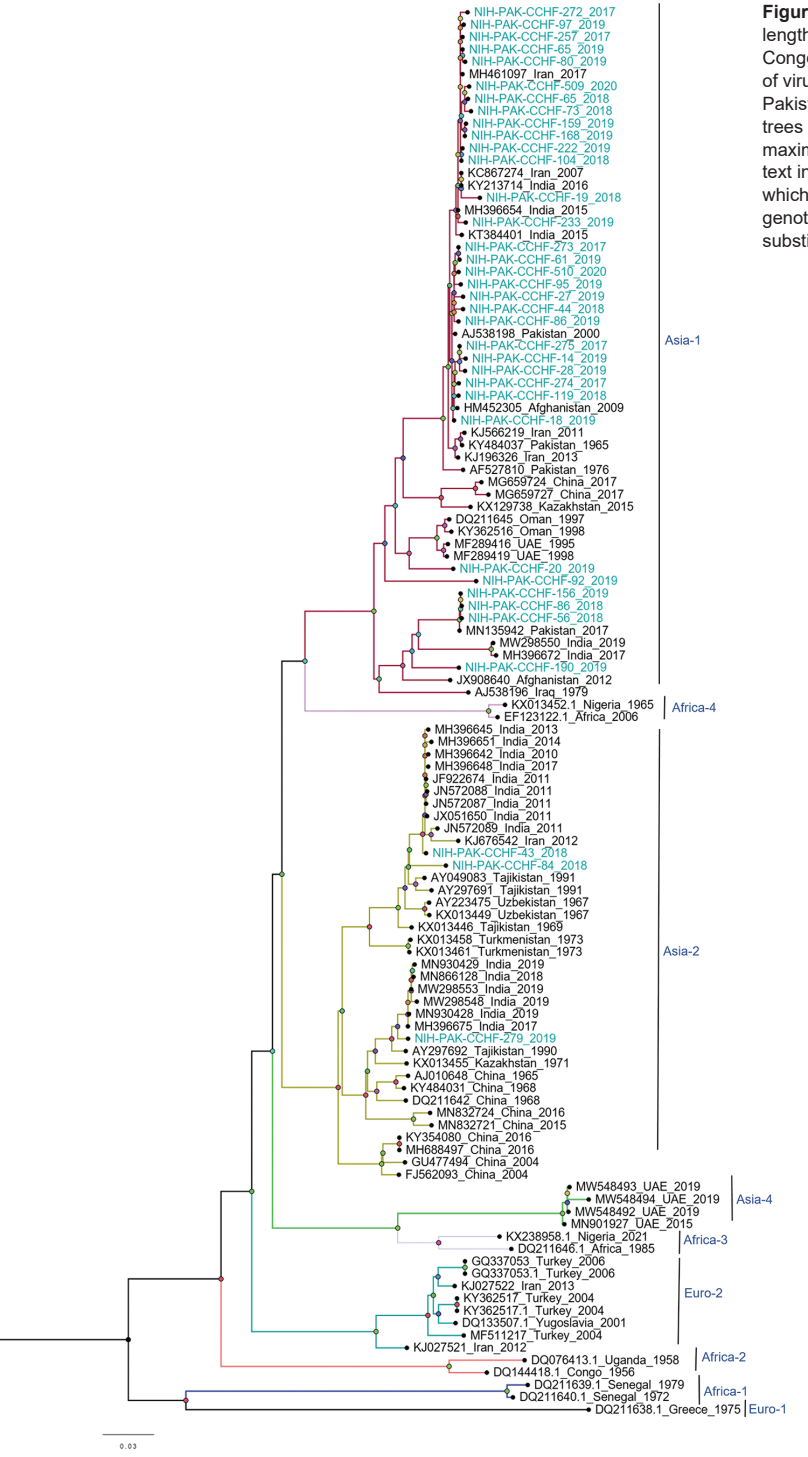
Phylogenetic analysis of full-length small gene segments of Crimean-Congo hemorrhagic fever virus in study of virus diversity and reassortment, Pakistan, 2017–2020. Midpoint-rooted trees were generated by using the maximum-likelihood method. Blue-green text indicates sequences from this study, which clustered with the Asia-1 and Asia-2 genotypes. Scale bar indicates nucleotide substitutions per site.

**Figure 4:**
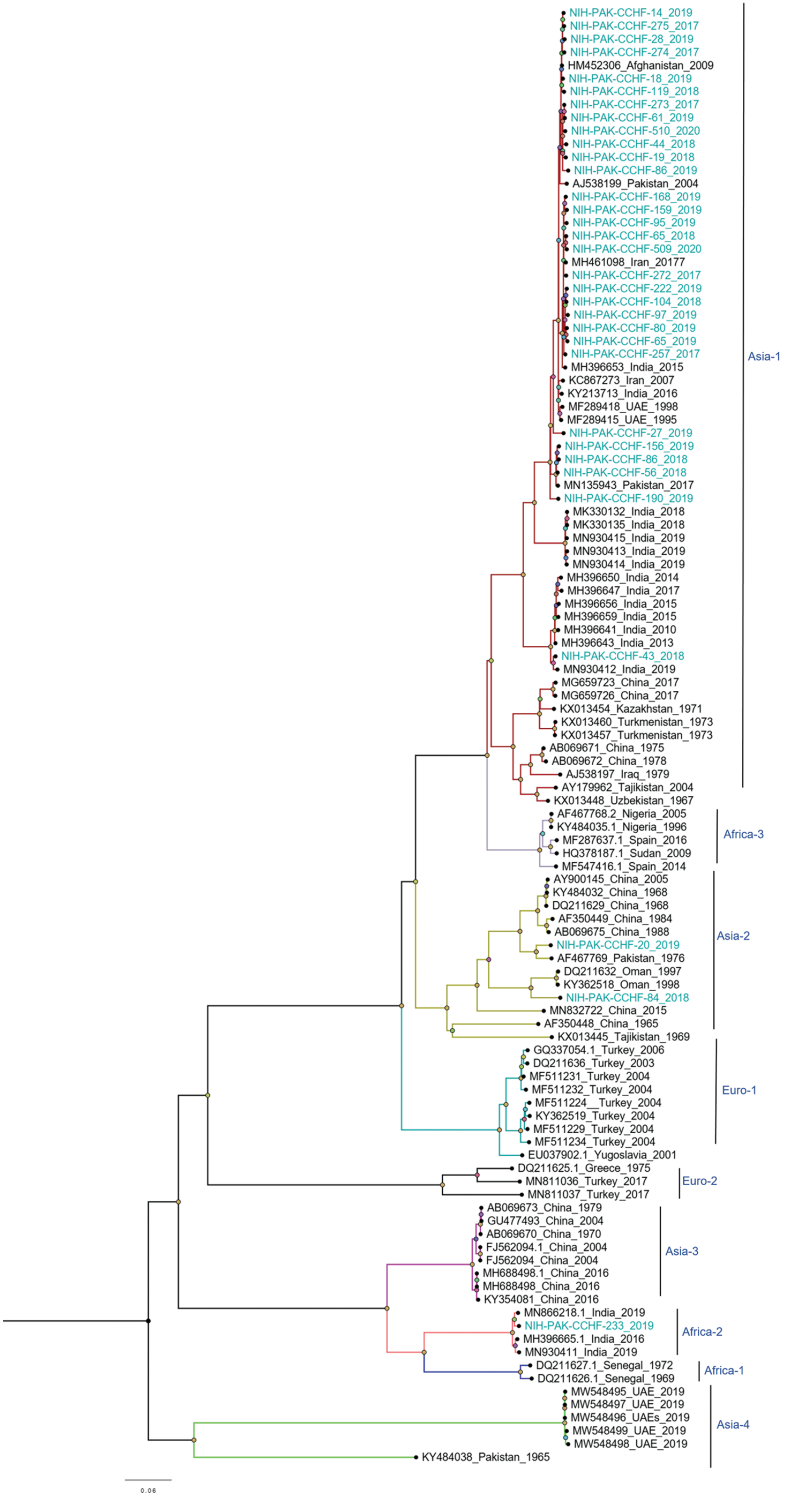
Phylogenetic analysis of full-length medium gene segments of Crimean-Congo hemorrhagic fever virus in study of virus diversity and reassortment, Pakistan, 2017–2020. Midpoint-rooted trees were generated by using the maximum-likelihood method. Blue-green text indicates sequences from this study. Most M segments from Pakistan clustered with the Asia-1 genotype/clade, but 3 reassorted sequences clustered with the Asia-2 clade, and 1 reassorted sequence clustered with the Africa-2 clade. Scale bar indicates nucleotide substitutions per site.

**Figure 5:**
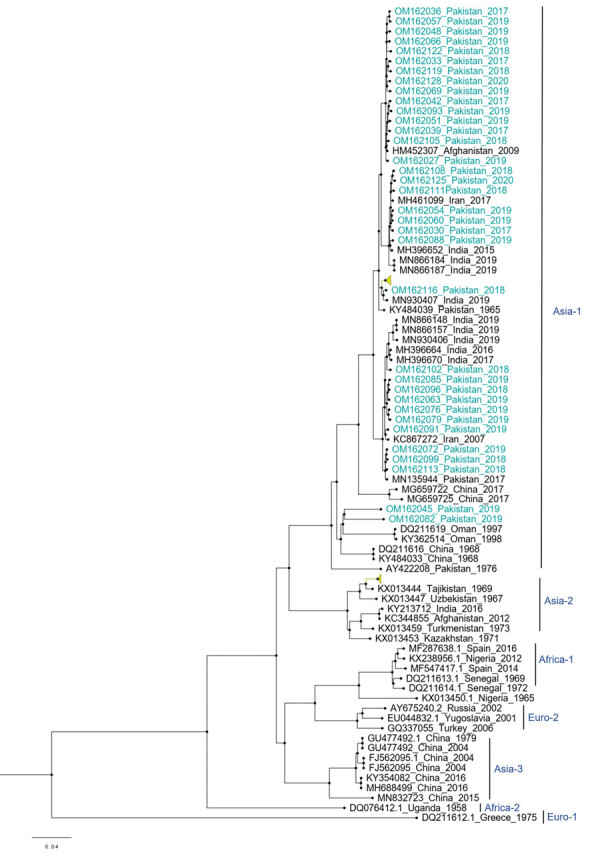
Phylogenetic analysis of full-length large gene segments of Crimean-Congo hemorrhagic fever virus in study of virus diversity and reassortment, Pakistan, 2017–2020. Midpoint-rooted trees were generated by using the maximum-likelihood method. Blue-green font indicates sequences from this study, which clustered only with the Asia-1 genotype. Scale bar indicates nucleotide substitutions per site.

Among the 36 sequenced CCHFV samples, we obtained complete sequences for all S segments, 35 L segments, and 33 M segments. Phylogenetic analysis of all 3 segments indicated the Asia-1 genotype (n = 29/33) was dominant. Moreover, we detected reassortment in 4 samples (Table 2). When phylogenetic analysis of individual genomic segments included additional samples, a similar picture emerged. Analysis of S segments revealed that most (n = 33) CCHFV sequences from this study clustered into the Asia-1 genetic lineage along with strains previously reported from humans (GenBank accession nos. AJ538198 [in 2000] and AF527810 [in 1976]) and ticks (accession nos. MN135942 [in 2017] and KY484037 [in 1965]) in Pakistan and other regional countries, such as Iran, India, Afghanistan, China, Oman, and United Arab Emirates. Three sequences clustered with the Asia-2 genotype, along with strains from India, Tajikistan, Uzbekistan, Turkmenistan, and China (Figure 3). The M segment sequences showed the greatest diversity with most samples clustering into the Asia-1 lineage along with strains from India (2015–2016), Iran (2007 and 2017), Afghanistan (2009), United Arab Emirates (1995 and 1998), and Pakistan (2004 and 2017) (Figure 4). The 2 Asia-2 isolates grouped with strains from China, Oman, and the Matin strain from Pakistan (GenBank accession no. AF467769). An M segment sequence from Rawalpindi (Gujrat state) in 2019 clustered with the Africa-2 lineage along with viruses reported from India during 2016–2019 (accession nos. MN866218, MH396665, and MN930411) (Figure 4). Phylogenetic reconstructions of the L segment revealed clustering with the Asia-1 lineage; closest matches were from India (2015–2019), Iran, China, Oman, Afghanistan, and previously reported strains from Pakistan isolated from ticks (accession nos. MN135944 [in 2017] and KY484039 [in 1965]) and a human (accession no. AY422208 [in 1976]) (Figure 5). According to analyses of L segments, all CCHFVs from this study shared 98%–100% identity at the nucleotide and amino acid levels. 

**Table 2:** Genotypes of Crimean-Congo hemorrhagic fever viruses isolated from patients in Pakistan, 2017–2020*

Sample nos.	Strain name	District	Province/region	Collection date	Genotype			GenBank accession nos.
					L	M	S	
1	NIH-PAK-CCHF-257_2017	Peshawar	Khyber Pakhtunkhwa	2017	Asia-1	Asia-1	Asia-1	OM162030–2
2	NIH-PAK-CCHF-272_2017	Peshawar	Khyber Pakhtunkhwa	2017	Asia-1	Asia-1	Asia-1	OM162033–5
3	NIH-PAK-CCHF-273_2017	Peshawar	Khyber Pakhtunkhwa	2017	Asia-1	Asia-1	Asia-1	OM162036–8
4	NIH-PAK-CCHF-274_2017	Peshawar	Khyber Pakhtunkhwa	2017	Asia-1	Asia-1	Asia-1	OM162039–41
5	NIH-PAK-CCHF-275_2017	Rawalpindi	Punjab	2017	Asia-1	Asia-1	Asia-1	OM162042–4
6	NIH-PAK-CCHF-104_2018	Khyber Agency	Khyber Pakhtunkhwa	2018	Asia-1	Asia-1	Asia-1	OM162096–8
7	NIH-PAK-CCHF-86_2018	Rawalpindi	Punjab	2018	Asia-1	Asia-1	Asia-1	OM162099–101
8	NIH-PAK-CCHF-84_2018	Islamabad	Islamabad	2018	Asia-1	Asia-2	Asia-2	OM162102–4
9	NIH-PAK-CCHF-119_2018	Islamabad	Islamabad	2018	Asia-1	Asia-1	Asia-1	OM162105–7
10	NIH-PAK-CCHF-65_2018	Multan	Punjab	2018	Asia-1	Asia-1	Asia-1	OM162108–10
11	NIH-PAK-CCHF-73_2018	Multan	Punjab	2018	Asia-1	NA	Asia-1	OM162111–2
12	NIH-PAK-CCHF-56_2018	Chakwal	Punjab	2018	Asia-1	Asia-1	Asia-1	OM162113–5
13	NIH-PAK-CCHF-43_2018	Rawalpindi	Punjab	2018	Asia-1	Asia-1	Asia-2	OM162116–8
14	NIH-PAK-CCHF-44_2018	Mianwali	Punjab	2018	Asia-1	Asia-1	Asia-1	OM162119–21
15	NIH-PAK-CCHF-19_2018	Multan	Punjab	2018	Asia-1	Asia-1	Asia-1	OM162122–4
16	NIH-PAK-CCHF-18_2019	Rawalpindi	Punjab	2019	Asia-1	Asia-1	Asia-1	OM162027–9
17	NIH-PAK-CCHF-20_2019	Jamshoro	Sindh	2019	Asia-1	Asia-2	Asia-1	OM162045–7
18	NIH-PAK-CCHF-27_2019	Lodhran	Punjab	2019	Asia-1	Asia-1	Asia-1	OM162048–50
19	NIH-PAK-CCHF-28_2019	DG Khan	Punjab	2019	Asia-1	Asia-1	Asia-1	OM162051–3
20	NIH-PAK-CCHF-80_2019	Rawalpindi	Punjab	2019	Asia-1	Asia-1	Asia-1	OM162054–6
21	NIH-PAK-CCHF-61_2019	Peshawar	Khyber Pakhtunkhwa	2019	Asia-1	Asia-1	Asia-1	OM162057–9
22	NIH-PAK-CCHF-65_2019	Gujrat	Punjab	2019	Asia-1	Asia-1	Asia-1	OM162060–2
23	NIH-PAK-CCHF-97_2019	Karachi	Sindh	2019	Asia-1	Asia-1	Asia-1	OM162063–5
24	NIH-PAK-CCHF-86_2019	Rawalpindi	Punjab	2019	Asia-1	Asia-1	Asia-1	OM162066–8
25	NIH-PAK-CCHF-95_2019	Lahore	Punjab	2019	Asia-1	Asia-1	Asia-1	OM162069–71
26	NIH-PAK-CCHF-156_2019	Chakwal	Punjab	2019	Asia-1	Asia-1	Asia-1	OM162072–4
27	NIH-PAK-CCHF-92_2019	DG Khan	Punjab	2019	NA	NA	Asia-1	OM162075
28	NIH-PAK-CCHF-159_2019	Bahawalpur	Punjab	2019	Asia-1	Asia-1	Asia-1	OM162076–8
29	NIH-PAK-CCHF-168_2019	Islamabad	Islamabad	2019	Asia-1	Asia-1	Asia-1	OM162079–81
30	NIH-PAK-CCHF-190_2019	Haripur	Khyber Pakhtunkhwa	2019	Asia-1	Asia-1	Asia-1	OM162082–4
31	NIH-PAK-CCHF-222_2019	Rawalpindi	Punjab	2019	Asia-1	Asia-1	Asia-1	OM162085–7
32	NIH-PAK-CCHF-233_2019	Rawalpindi	Punjab	2019	Asia-1	Africa-2	Asia-1	OM162088–90
33	NIH-PAK-CCHF-279_2019	Islamabad	Islamabad	2019	Asia-1	NA	Asia-2	OM162091–2
34	NIH-PAK-CCHF-14_2019	Karachi	Sindh	2019	Asia-1	Asia-1	Asia-1	OM162093–5
35	NIH-PAK-CCHF-509_2020	Rawalpindi	Punjab	2020	Asia-1	Asia-1	Asia-1	OM162125–7
36	NIH-PAK-CCHF-510_2020	Peshawar	Khyber Pakhtunkhwa	2020	Asia-1	Asia-1	Asia-1	OM162128–30

*Genotypes were determined by whole-genome sequencing of L, M, and S virus segments. L, large; M, medium; NA, not applicable; S, small.

The L and S segments from the Africa-2 isolate from Pakistan in this study (NIH-PAK-CCHF-233_2019) belonged to the Asia-1 lineage, suggesting reassortment. In addition, 3 other isolates showed reassortment. The S and M segments of NIH-PAK-CCHF-84_2018 clustered with the Asia-2 genotype, and the L segment clustered with Asia-1. The L and M segments of NIH-PAK-CCHF-43_2018 had Asia-1 genotypes, whereas the S segment had an Asia-2 genotype. Similarly, the S and L segments of NIH-PAK-CCHF-20_2019 had Asia-1 genotypes, and the M segment clustered with the Asia-2 genotype.

## Discussion

CCHF is endemic in Pakistan, and data reported since 2010 indicate an increase in human CCHF cases. The first human CCHF case was reported in 1976, and several sporadic outbreaks have been reported since then from different parts of the country (*13*,*16*). Human CCHF-positive case counts have increased during 2011–2020; a total of 605 confirmed CCHF cases have been reported (*17*–*19*). The increase in cases can be attributed to multiple factors, including the country’s underdeveloped healthcare system that has insufficiently trained healthcare professionals and lacks equipment to manage CCHF, as well as an insufficient number of healthcare facilities that offer CCHF management. Furthermore, the general public is relatively uninformed about CCHFV vector control and risks for transmission to healthy persons while handling livestock and conducting animal husbandry. CCHFV transmission risk becomes higher in urban areas during the Eid ul Azha festival, which includes ritual animal slaughter. 

The porous border between Pakistan and Afghanistan has large refugee and nomadic tribal movements, often accompanied by their cattle, across Balochistan Province and Khyber Pakhtunkhwa Province, which also contribute to CCHFV transmission; Afghanistan is also a CCHF-endemic country (*20*). According to the NIHP, 296 cases of CCHF were reported during 2015–2020; most infections were from Balochistan Province (39%), then Punjab (24%), Khyber Pakhtunkhwa (14%), Sindh (12%), and Islamabad (5%) (*18*). Balochistan and Punjab Provinces contributed most of the CCHF cases. Khyber Pakhtunkhwa and Balochistan Provinces border CCHF-endemic countries; Khyber Pakhtunkhwa Province shares a border with Afghanistan, and Balochistan Province shares a border with Iran, where livestock movement takes place routinely and, thus, potentially contributes to reported CCHF case numbers (*13*,*21*). Animal surveillance studies from Pakistan have reported CCHFV from all over Pakistan but primarily from the Balochistan region. Less CCHFV cases have been reported from Punjab Province, although it harbors the largest animal population because of greater agricultural land mass (*13*,*22*,*23*), which could potentially favor the tick–vertebrate–tick life cycle. 

*Hyalomma* and *Rhipicephalus* spp. (Ixodidae family) ticks, reported as the most prevalent tick species in southern, western, and northern Punjab (*24*,*25*), are the main spreaders of CCHFV in different regions of Pakistan and increase the risk for CCHF outbreaks. Regions of upper Punjab, such as Chakwal, Mianwali, Rawalpindi and Attock, have >20% prevalence of ticks with CCHFV, compared with the lower Punjab regions of Rajanpur and Lahore, which have <10% prevalence. *Hyalomma* and *Rhipicephalus* ticks infesting livestock have been reported from Balochistan Province, where the district of Kalat has the largest percentage of CCHFV-positive ticks (60%), followed by Quetta (30%) and Qilla Abdullah (10%). The semiarid climate comprising shrub rangelands in Balochistan Province appears to favor tick growth (*13*,*23*). Furthermore, Punjab Province, particularly in the upper region, has large rangelands for animal grazing, a semiarid climate with high precipitation, and abundant livestock, which also provide a thriving tick habitat and can subsequently increase CCHFV prevalence (*26*). Previous studies have reported high CCHFV IgG seroprevalence in humans from Upper Punjab (*27*,*28*), which suggests effective control measures are especially needed in this area to inhibit tick infestation of livestock and prevent CCHF outbreaks.

The lack of next-generation sequencing capabilities in Pakistan has been a major limitation for determining the genomic diversity of circulating CCHFV. Previously, partial sequencing of the S gene segment was used for classification and phylogenetic analysis of CCHFV (*13*,*16*,*29*–*31*). Because the S segment is conserved and relatively short compared with L and M segments, it was used as a surrogate for performing phylogenetic analysis. Evidence of reassortment (*31*–*34*) among CCHFV genomes confirmed the need for whole-genome or partial sequencing of all 3 gene segments (*35*). CCHFV samples collected from infected patients intermittently during 1965, 1976, and 2000–2002 in Pakistan showed the prevalence of the Asia-1 genotype and phylogenetic association with viruses from the neighboring countries of Iran, Afghanistan, and United Arab Emirates (*31*). During 2008–2011, the Asia-1 genotype was the most prevalent lineage circulating in the southwest region of Pakistan, specifically Balochistan Province, which borders the CCHF-endemic countries of Iran and Afghanistan (*13*,*17*). Moreover, in 2008, a single case of Asia-2 genotype was also reported from Quetta in Balochistan Province, which was phylogenetically related to viruses from Uzbekistan, Tajikistan, and Kazakhstan (*29*). According to S segment sequencing, another study on CCHFV in Pakistan during 2019 involved 14 districts and further corroborated the endemicity of the Asia-1 genotype (*30*). In this study, most (80.5%, n = 29) CCHFVs circulating in Pakistan clustered with the Asia-1 (S, M, and L segments) clade alongside strains from neighboring (Iran, India, Afghanistan, and China) and regional (Oman and United Arab Emirates) countries, indicating CCHFV transmission between those countries. Circulation of genetically similar CCHFV strains has been reported in Iran, where frequent animal trade has been hypothesized to cause CCHFV movement between Pakistan and neighboring countries (*13*,*36*–*39*).

In this study, we observed 4 (11%) reassortment events among the 36 whole-genome sequences. Although frequent reassortment events have been reported in the M segment, rendering high virus fitness, we observed reassortment in the S segment as well. The Matin strain isolated from Pakistan has been a good example of CCHFV reassortment events in this region (*15*). Segmental reassortment in RNA viruses has been associated with emergence of pandemic virus strains and antigenic shifts (*33*). The Africa-2 reassorted virus (NIH-PAK-CCHF-233_2019) shared a common clade with sequences from India isolated from pooled tick specimens. Two of the sequences from India (GenBank accession nos. MN866218 and MN930411) were isolated from ticks from Rajasthan and Gujarat state in 2019, showing 98% homology at the nucleotide and amino acid levels with the Africa-2 isolate from Pakistan. Rajasthan state in India shares a border with Punjab and Sindh Provinces of Pakistan, but Gujarat state is more centrally located and does not share a border with Pakistan. M segments of the Asia-2 genotype also clustered with sequences isolated from ticks from China (GenBank accession no. MN832722.1). In China, the virus has been isolated from *Hyalomma asiaticum* ticks. However, in Pakistan, Iran, Turkmenistan, and Tajikistan, *H. anatolicum* is the main CCHFV vector (*40*). Furthermore, both *H. anatolicum* and *Rhipicephalus* ticks have been reported as the primary vectors of CCHFV in Pakistan and Iran (*41*). Because of the diversity of ticks in different regions, further investigation of CCHFV prevalence in various tick species in Pakistan is needed. 

Surveillance of CCHFV reassortment, although difficult, is crucial for health authorities. Whether reassortments can be linked to increased virus pathogenicity or disease severity requires further study. Variations in antigenicity among CCHFV isolates have not been reported but need to be considered for future vaccines (*32*). Rapid diagnostics for identifying and managing outbreaks are pivotal; however, considering the evolutionary dynamics of CCHFV strains, immunological assays should be used in conjunction with PCR to achieve high diagnostic sensitivity. A concurrent need exists for better understanding of CCHFV antibody kinetics in clinically diverse samples, and next-generation sequencing can help identify mutant viruses.

In conclusion, we identified CCHFV sequences with verifiable genomic reassortments and highlight the importance of sequencing all 3 virus segments. Our results suggest diversification of circulating strains of CCHFV in Pakistan and warrant rigorous surveillance and follow-up of CCHF cases, particularly in disease-endemic regions of the country. The CCHFV Asia-1 genotype has been prevalent in Pakistan, but the Africa-2 genotype might also be emerging. CCHFV sequences from this study are from humans; however, sequences from other host vertebrates, particularly from ticks, will also be required to identify CCHFV mutations and evolutionary dynamics in different regions of the world. Because CCHFV reassortment is a continual evolutionary phenomenon, which can genetically shift virus genomes enhancing pathogenicity, careful application of routine and effective control measures that reduce overall tick abundance in the environment can likely bring substantial reduction in risk for CCHFV transmission to humans. Furthermore, genomic surveillance of CCHFV is needed to identify the major circulating genotypes in Pakistan and elsewhere, which will further aid in containment of disease.
